# Real-time monitoring of mitochondrial oxygenation during machine perfusion using resonance Raman spectroscopy predicts organ function

**DOI:** 10.21203/rs.3.rs-3740098/v1

**Published:** 2023-12-21

**Authors:** Rohil Jain, Emmanuella O. Ajenu, Ehab O.A. Hafiz, Padraic Romfh, Shannon N. Tessier

**Affiliations:** Harvard Medical School & Massachusetts General Hospital; Harvard Medical School & Massachusetts General Hospital; Theodor Bilharz Research Institute; Pendar Technologies; Harvard Medical School & Massachusetts General Hospital

## Abstract

Organ transplantation is a life-saving procedure affecting over 100,000 people on the transplant waitlist. Ischemia reperfusion injury is a major challenge in the field as it can cause post-transplantation complications and limits the use of organs from extended criteria donors. Machine perfusion technology is used to repair organs before transplant, however, currently fails to achieve its full potential due to a lack of highly sensitive and specific assays to predict organ quality during perfusion. We developed a real-time and non-invasive method of assessing organ function and injury based on mitochondrial oxygenation using resonance Raman spectroscopy. It uses a 441 nm laser and a high-resolution spectrometer to predict the oxidation state of mitochondrial cytochromes during perfusion, which vary due to differences in storage compositions and perfusate compositions. This index of mitochondrial oxidation, or 3RMR, was found to predict organ health based on clinically utilized markers of perfusion quality, tissue metabolism, and organ injury. It also revealed differences in oxygenation with perfusates that may or may not be supplemented with packed red blood cells as oxygen carriers. This study emphasizes the need for further refinement of a reoxygenation strategy during machine perfusion that is based on a gradual recovery from storage. Thus, we present a novel platform that provides a real-time and quantitative assessment of mitochondrial health during machine perfusion of livers, which is easy to translate to the clinic.

## Introduction

Currently, over 100,000 individuals anxiously await a lifesaving organ transplant, yet thousands of these will die every year while on the waitlist due to a severe organ shortage. Current clinical standards for organ handling for transplantation include a period of cold ischemia during transport, which is aimed at suppressing metabolism to extend *ex vivo* graft survival by several hours. Human livers, for instance, can be stored for up to 8 hours in static cold storage (SCS) immersed in a preservation solution without additional dissolved oxygen^1,2^. Following cold ischemia, grafts are then exposed to a sudden burst of oxygen supply during implantation, resulting in ischemia-reperfusion injury (IRI). IRI is characterized by a reduction in the ATP levels, accumulation of metabolic waste products^3,4^, and production of reactive oxidative species (ROS), which damages the vascular endothelium and activates a cascade of events that may lead to cell death and organ dysfunction^5,6^. IRI remains a significant challenge in transplantation that is responsible for posttransplant graft dysfunction^7,8^ and is poised to limit the utilization of grafts that are exposed to warm ischemic injury, including donation after cardiac death (DCD)^9,10^.

Several strategies currently exist to mitigate IRI with the goal of reducing (or eliminating) ischemia while minimizing reperfusion injury. *Ex situ* machine perfusion (MP) is currently the leading strategy that maintains organs in an active state and delivers oxygen and nutrients via a perfusate prior to transplantation. The metabolic activity and oxygen demand during MP can vary considerably depending on the temperature of perfusion. Simultaneously, different perfusate mediums can be altered to supply the desired amounts of oxygen and nutrients. For example, Normothermic Machine Perfusion (NMP), an FDA approved protocol, involves maintaining the organ at a physiological temperature of 37°C and supplementing the perfusate with an oxygen carrier, such as packed red blood cells (pRBC)^11^ or synthetic hemoglobin from swine (HBOC201^19^) and murine sources (Hemarina M101^20^). While NMP minimizes ischemia time, grafts are continuously maintained in a hyper-oxygenated state^12^. Sub-normothermic machine perfusion (SNMP) is another popular method that maintains organs at 21°C with an acellular perfusate carrying only dissolved oxygen that should be sufficient to meet lower metabolic demands. Like NMP, SNMP reduces ischemia; however, uses a more gradual increase in metabolism that may protect injured organs^13^ from a sudden burst in oxygen consumption at 37°C^14^. Finally, hypothermic oxygenated machine perfusion (HOPE) uses dissolved oxygen in a perfusate pumped at 4–10°C as a means to prevent early mitochondrial injury upon reperfusion^15,16^, although it represents a departure from physiology.

Despite the protective benefits of these techniques, mechanistic insights into IRI and the optimal oxygenation strategy to improve metabolic recovery while minimizing ROS injury during machine perfusion is lacking in the field. Mitochondria are attractive targets to observe reoxygenation due to their extensive role in metabolism and IRI^27–29^. Measurement of mitochondrial activity can be used to study energetic recovery via ATP production after ischemia and reperfusion. Further, injury to mitochondria may also predict cellular damage. Despite the importance of mitochondria, current technologies to measure mitochondrial function during machine perfusion lack sensitivity/specificity, do not capture the dynamic nature of oxygenation as a function of IRI, or rely on destructive tissue sampling. Instead, we developed a metric of mitochondrial function using a real-time, non-destructive measurement of mitochondrial redox state using resonance Raman spectroscopy (RRS)^30^.

The reduced and oxidized states of mitochondrial cytochromes have unique resonance Raman spectral signatures when excited with a 441 nm laser. This spectrum is recorded from the surface of organs during MP, and simultaneously cross referenced with fully oxidized and reduced mitochondrial cytochrome spectral libraries in real-time, to derive the resonance Raman reduced mitochondrial ratio (3RMR). 3RMR is defined as a ratio of reduced to total mitochondrial complex redox states. In a healthy condition there is a steady transfer of electrons from the mitochondrial complexes to oxygen, which keeps them in an oxidized state, i.e., with low 3RMR. However, such transfer can be disrupted during ischemia and reperfusion and lead to a higher reduced state, i.e., higher 3RMR. In contrast to other methods, the 3RMR level can be used to directly measure metabolic utilization of oxygen for ATP production with high sensitivity and specificity. Moreover, it works independent of the presence of hemoglobin yet, provides complementary hemoglobin saturation measurement; and thus, can be used with both RBC-based and acellular perfusates. Finally, it is a non-invasive and real-time assay free of tissue biopsies and cumbersome mitochondrial isolation procedures^31^, thus making it an ideal platform for assessment during machine perfusion.

To leverage this powerful technology to understand IRI and develop and assay of mitochondrial health, we measured 3RMR during normothermic machine perfusion of rodent livers that were exposed to minimal versus extended cold ischemia durations (0-hour cold ischemia vs 24-hour cold ischemia). We also varied the NMP protocol to include either an acellular perfusate or packed RBCs to understand the impact of oxygen delivery methods on reperfusion injury. As expected, the 3RMR values reflected the oxygen consumption and metabolism patterns consistent with the expected trends for each storage condition and oxygenation strategy. The red blood cells provided sufficient oxygen supply to support the starving organ after 24 hours of ischemia, however dealt higher endothelial damage. On the other hand, the acellular perfusate provided insufficient oxygen after ischemia but treated the endothelial layer gently. We also support these observations with other markers of perfusion and metabolic recovery as well as injury, highlighting the robustness of this index in predicting mitochondrial health and organ recovery.

## Results

### RRS allows quantification of mitochondrial redox state with different perfusate compositions.

**Figure 1:**
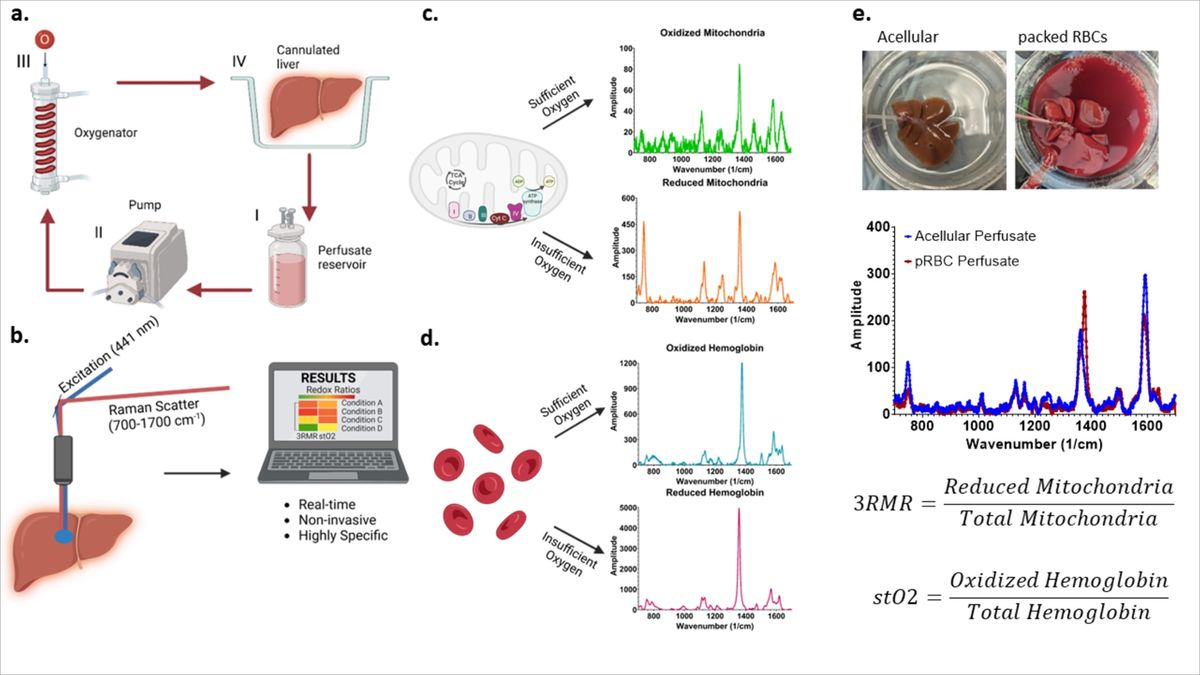
Resonance Raman Spectroscopy predicts functional oxygenation of liver tissue during machine perfusion. **a**, schematic of machine perfusion of livers where a perfusate(I) with or without red blood cells as oxygen carriers is circulated by a pump (II) to an oxygenator (III). The oxygenator consists of a gas-permeable tubing that carries the perfusate surrounded by oxygen at a higher ambient partial pressure than atmosphere. This allows the perfusate to be oxygenated before it is supplied to a cannulated liver (IV). **b,** the principle of our custom approach to measuring mitochondrial redox state in the tissue non-invasively using a resonance Raman spectroscopy device. A 441nm excitation laser is used to excite molecules with a porphyrin ring (such as mitochondrial complexes, cytochromes, and hemoglobin) that produce a resonance Raman spectrum. **c,** shows the spectrum of oxidized and reduced isolated mitochondria. **d,** shows the spectrum of reduced and oxidized hemoglobin. **e,** shows two rat livers that are sitting in the perfusion bowl that are supplied with oxygen either with or without RBCs. Directly below the livers are the RRS spectrum from each liver. This spectrum is deconvoluted into its constituent molecular signatures using pre-recorded libraries as shown in c and d. The deconvolution coefficients for the reduced mitochondria is averaged with the sum of reduced and oxidized mitochondria to quantify the redox state of tissue and called the 3RMR value. Similarly, the oxygen saturation of hemoglobin in the tissue is also obtained by taking the ratio of oxidized hemoglobin coefficient with the total coefficients of oxidized and reduced hemoglobin.

RRS allows accurate quantification of the redox state of mitochondria as well as hemoglobin in the perfusate since both mitochondrial complexes and hemoglobin molecules possess a porphyrin ring structure with a strong Soret absorption band at 441 nm wavelength. Excitation near the Soret band results in a resonant enhancement of the vibrational modes that result in Raman spectra. When excited by this laser during machine perfusion (Fig. 1a), the vibrational scattering spectrum from the liver surface is carried via optical fibers to a Charge Coupled Device (CCD) array, and eventually analyzed using custom LabVIEW program (Fig. 1b). This program deconvolves the spectrum into its components by using pre-recorded libraries of fully reduced and oxidized mitochondrial complexes and hemoglobin (Fig. 1c, d) by minimizing error in a statistical regression fit. Depending on the perfusate composition, either only the mitochondrial libraries, or both mitochondrial and hemoglobin libraries are used for calculating 3RMR. Upon deconvolution, weights are assigned to each complex which are then used to calculate the Resonance Raman Reduced Mitochondrial Ratio (3RMR) which is the ratio of the weights of reduced to total mitochondrial 3RMR (Fig. 1e)^30^. Figure 1e also shows representative images of rodent liver perfusions with and without the packed RBCs, and representative spectrum from each type of perfusion. A more detailed description of the perfusion system and the Raman device is available in the methods section.

### 3RMR reflects oxygenation dynamics and energetic recovery of 0-hour cold ischemic & 24-hour cold ischemic livers in real time during perfusion.

**Figure 2:**
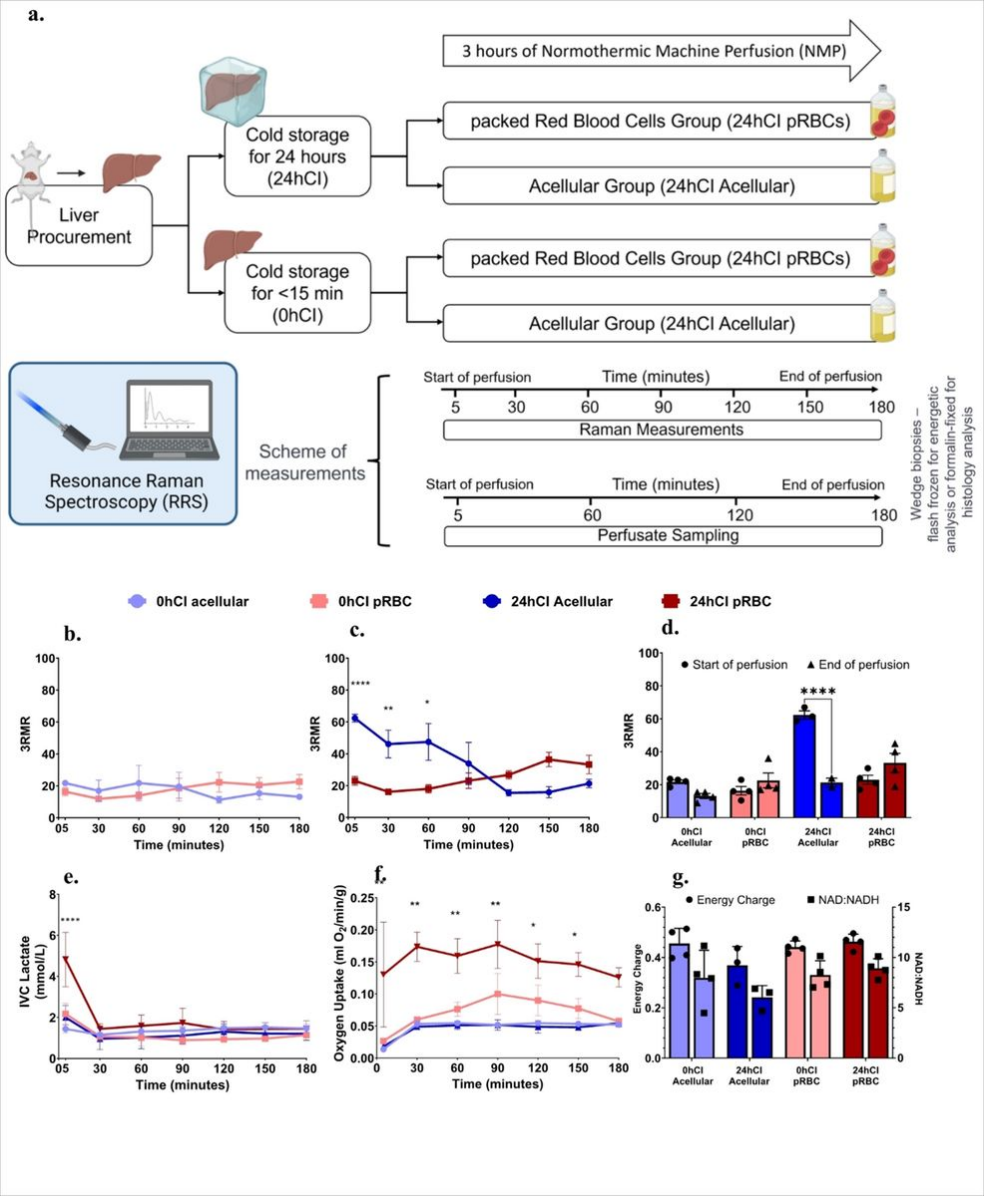
3RMR with lactate, energy charge, and NAD:NADH ratio predict metabolic recovery dynamics during machine perfusion. **a,** Schematic of the experiments and test groups. Each liver is either stored at 4°C for 24 hours or perfused immediately after recovery from the rat. It may then be machine perfused with either a packed RBC based perfusate or an acellular perfusate for 3 hours at 37°C. During this time the perfusate is sampled every 60 minutes for blood gas, blood chemistry, and ALT/AST analysis. Raman measurements are also taken from the surface of the livers every 30 minutes. At the end of the perfusion, wedge biopsies from the liver are either flash frozen for energetic tests or stored in formalin for histology. **b,** shows low 3RMR values throughout the 3 hours of perfusion that are statistically indistinguishable between 0hCI acellular and 0hCI pRBC groups. **c,** shows significantly higher 3RMR value immediately after reperfusion of 24hCI acellular group compared to 24hCI pRBC group. The 3RMR value decreases continuously during perfusion and becomes statistically indistinguishable around 90 minutes of perfusion. This trend reverses after 90 minutes indicating possible dysfunction of mitochondrial electron transport chain, however remaining statistically the same. **d,** shows that the comparison between 3RMR values at the start of perfusion and at the end of perfusion for each of the tested conditions. The p-values that are marked for each pair show statistically significant lower 3RMR values for acellular perfusate while those with packed RBCs are statistically the same. **e,** shows lactate levels during the 3 hours of perfusion for all four conditions. that remain low and indistinguishable for all the tested conditions during perfusion. **f,** shows oxygen uptake rate for all four groups during perfusion. The oxygen uptake for 24hCI pRBC group is the highest which reflects the higher oxygen demand that is satisfied by the higher supply of oxygen bound to RBCs. OUR between the other three groups is comparable with only slightly higher values in the 0hCI pRBC group. **g,** shows energy charge and NAD:NADH values for all the four conditions tested. These were obtained from flash frozen wedge biopsies from the livers at the end of 3 hours of perfusion. While not statistically significant, the ratios for machine perfusion of 24-hour cold ischemic livers perfused with acellular perfusate were slightly lower than the other conditions, indicating potential slower recovery of oxidative phosphorylation. Statistical significance levels - * p<0.033, ** p<0.0021, *** p<0.0002, **** p<0.0001.

Our study to observe ischemia and reoxygenation during machine perfusion using 3RMR consisted of groups that reflect different degrees of ischemic stress in the form of short or long durations of storage at 4°C – one group of livers had minimal ischemia (< 15 minutes), referred to as the 0 hour cold ischemic (0h-CI) livers; and a second group of livers that were stored for 24 hours in cold ischemic condition (24h-CI) livers. These groups were chosen because the former mimics fresh transplantations with minimal ischemia, while latter mimics the longest cold ischemic duration that maintains 100% survival after transplantation based on previous studies by our group^32^. We also further tested two perfusate compositions – one that contained packed RBCs as oxygen carriers (pRBC group) and a second group where no oxygen carriers were used (acellular group). These perfusate compositions were chosen to understand the effects of different levels of oxygen stress and test the usefulness of the RRS device in each condition. A summary of the storage and perfusion conditions is shown in Fig. 2a. It was expected that the 24h-CI livers would be starved for oxygen and benefit from high oxygen supply, however, also experience higher IRI. 0h-CI livers on the other hand, are expected to experience a lower basal metabolic demand for oxygen and experience minimal IRI.

As shown in Fig. 2b, we observed a low 3RMR value for 0h-CI livers irrespective of the mode of oxygenation. At the start of perfusion, the acellular perfusate group showed a 3RMR of 21.75 ± 2.25, and the pRBC group showed a 3RMR of 16.38 ± 5.121 which were statistically similar (p = 0.1031). The values remained low throughout the 3 hours of perfusion, with acellular group 3RMR 13.13 ± 3.09, and pRBC 3RMR 22.63 ± 8.97 (p = 0.09) at the end of perfusion. This shows sufficient oxygenation with both perfusates, indicating that the dissolved oxygen at a higher partial pressure (500–600 mm Hg) is sufficient for meeting the oxygen demand of these livers. However, this was different from the trend in 24h-CI group of livers, as shown in Fig. 2c. The 3RMR value was high immediately upon reperfusion in the 24h-CI acellular group with a mean of 62.33 ± 4.25, which was in contrast with the low 3RMR in the 24hCI-pRBC group at 23 ± 5.48 (p-value = 0.0002). The high 3RMR likely indicates a high demand of a starving liver which is insufficiently satisfied by the acellular perfusate carrying lower oxygen than the pRBC perfusate, however, the exact mechanism for observation of reduced cytochromes remains to be studied. Interestingly, the 3RMR for 24hCI-acellular livers decreases during perfusion to 34.22 ± 22.48 at 90 minutes, which becomes statistically indistinguishable (p = 0.9426) from the 24hCI-pRBC group by the end of perfusion (33.25 ± 11.62). This appears consistent with the observation that 24 hour cold stored livers can be fully recovered with machine perfusion^33^. The 3RMR values at the beginning and the end of perfusions for each group are also summarized in Fig. 2d highlighting significant improvement in the 24h-CI acellular group.

The initial period of hypoxia and subsequent recovery of 24h-CI groups was also reflected in other perfusion-based biochemical markers of metabolism. Figure 2e shows the high venous lactate concentration in the 24hCI-pRBC group (4,828 ± 2.64 mM) compared to 24h-CI acellular (2 ± 1 mM), 0h-CI pRBC (2.175 ± 1.026 mM) and 0h-CI acellular (1.44 ± 0.39 mM) groups. This may occur due to lactate production in the red blood cells or from liver cells as a result of high oxygen starvation due to long duration of cold ischemia. The lactate buildup in 24h-CI pRBC group gets cleared (1.44 ± 0.5 mM) by the first 30 minutes of perfusion and becomes statistically similar to 24hCI-acellular (0.95 ± 0.9 mM) and 0hCI-pRBC (1.07 ± 0.41 mM) groups (p = 0.404 & p = 0.302 respectively). This trend of low lactate values continues until the end of 3 hours of perfusion when the lactate values are as follows: 0h-CI acellular-1.47 ± 0.425 mM; 0h-CI pRBC-1.14 ± 0.118 mM; 24h-CI acellular – 1.203 ± 0.546 mM; 24h-CI pRBC- 1.45 ± 0.764 mM. Figure 2f shows oxygen uptake rate (OUR) by livers in all groups. Here, we observe higher oxygen uptake by the 24hCI-pRBC group compared to other groups in the initial phase of perfusion- for instance, the oxygen uptake rate at the beginning of perfusion were as follows for the following groups-0hCI acellular, 0hCI pRBC, 24hCI acellular, and 24hCI pRBC: 0.014 ± 0.002, 0.027±, 0.018 ± 0.005, and 0.13 ± 0.164 ml/min respectively (p = 0.97 0hCI pRBC vs 0hCI acellular; p = 0.0082 24hCI pRBC vs 24hCI acellular); and 90 minutes were 0.052 ± 0.004 ml/min, 0.1 ± 0.064 ml/min, 0.052 ± 0.014 ml/min, and 0.177 ± 0.075 and ml/min respectively (p = 0.9, for 0hCI pRBC vs acellular; p = 0.0116 for 24hCI pRBC vs acellular). However, levels became lower to statistically similar level as all other groups by the end of perfusion with OUR as follows at 3 hours of perfusion: 0.052 ± 0.005 ml/min, 0.057 ± 0.011 ml/min, 0.054 ± 0.01 ml/min, and 0.126 ± 0.03 ml/min respectively ((p = 0.99, for 0hCI pRBC vs acellular; p = 0.16 for 24hCI pRBC vs acellular). The lower OUR during perfusion in the 24hCI acellular group compared to 24hCI pRBC group likely reflects limited oxygen availability and causes some damage to the liver. Finally, Fig. 2g shows energy charge (EC), defined as ratio (ATP + 0.5*ADP) / (ATP + ADP + AMP) ^34^ and NAD:NADH ratio for all groups. Energy charge is an indicator of energetic recovery at the end of perfusion, while NAD:NADH ratio is an indicator of utilization of substrates at complex I of the electron transport chain during machine perfusion. EC shows statistically similar levels in all the groups of livers at end of the 3 hours of perfusion- 0hCI acellular-0.455 ± 0.06, 0hCI pRBC- 0.442 ± 0.024, 24hCI acellular 0.369 ± 0.075, and 24hCI pRBC- 0.462 ± 0.03 (p = 0.99 for all comparisons). This may indicate a restoration of ATP across all experimental groups. On the other hand, NAD:NADH ratios for each group were 7.98 ± 2.74, 8.25 ± 1.41, 6.03 ± 1.19, and 8.92 ± 0.96, respectively with p > 0.05 for all pairwise comparisons, except 24hCI acellular vs 24hCI pRBC where the difference between groups was statistically significant with p = 0.029. Based on these metabolic markers, it may be hypothesized that 3RMR, ATP, and NAD:NADH ratios confirm energetic recovery for all groups of livers, albeit sub-optimally in the 24hCI acellular perfusion group where the livers may be experiencing minimal levels of hypoxia due to an incomplete recovery from ischemic stress.

### Increased levels of injury markers indicate suboptimal recovery of livers perfused with pRBCs

**Figure 3:**
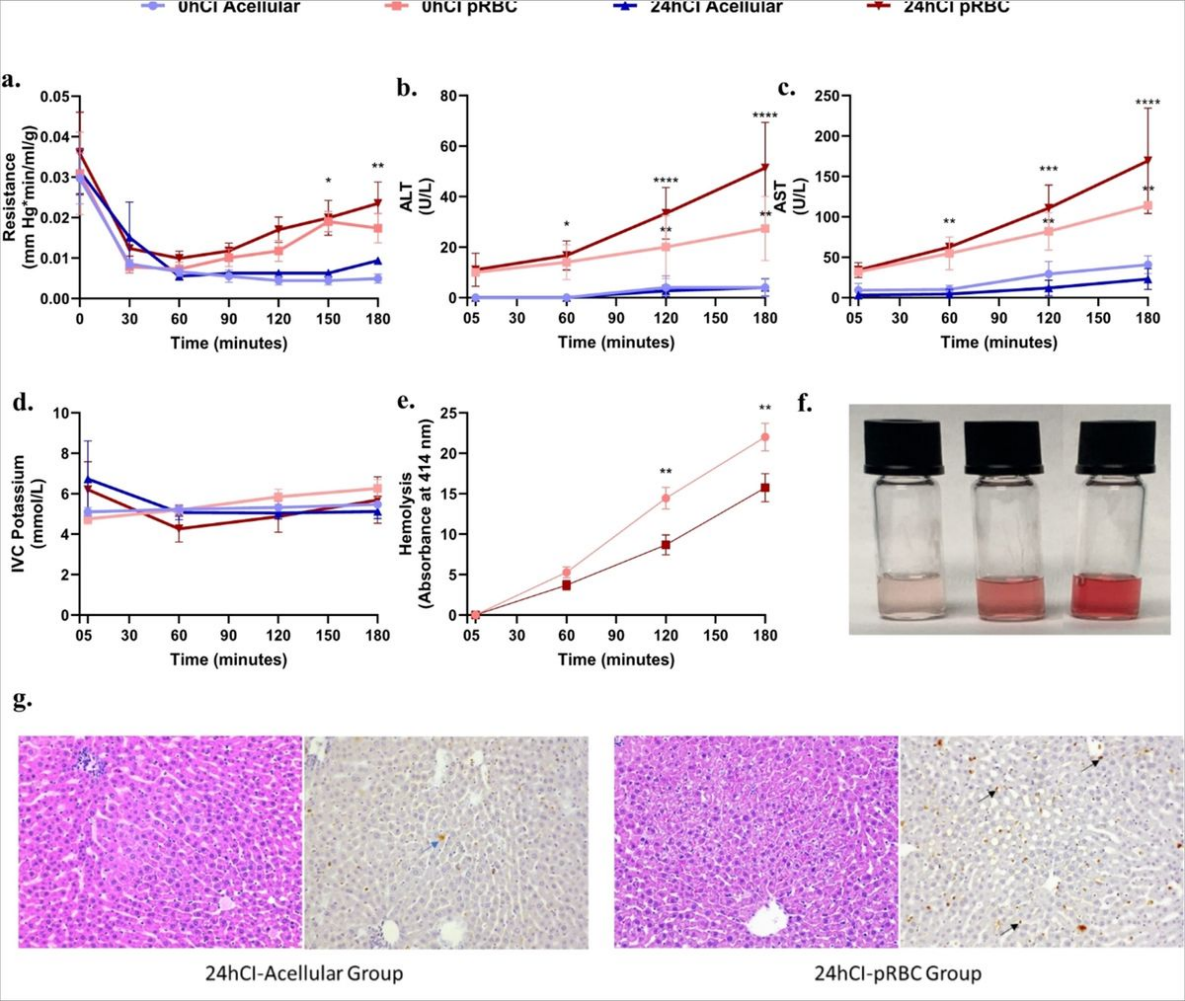
Portal vein pressure, ALT, and AST levels indicate higher injury to livers perfused with packed RBCs compared to acellular perfusate for the 3 hours of perfusion. **a,** Shows the trends in resistance for the tested conditions. It is significantly higher in the pRBC groups than the acellular perfusate groups after 2 hours of perfusion. There is no significant difference between the fresh and cold storage groups for each type of perfusate. **b,c,** Show trends in ALT and AST levels between the four groups, where pRBC perfusate groups show higher values compared to the acellular perfusate groups. The statistically significant difference becomes more prominent over time for these groups. **d,**Shows no significant difference in IVC potassium for all four groups during perfusion. **e,** Shows hemolysis over the duration of perfusion. There is no significant difference at any time during the perfusion between 0hCI and 24hCI groups. The hemolysis is also evident from the increasing red coloration of the perfusate samples with time as shown in **f.** Finally, **g,** shows representative histology to show the patters of injury in 24hCI livers from acellular and pRBC groups stained with H&E and TUNEL stains (Magnification 20x). The blue arrows indicate hepatocyte death while the black arrows show endothelial death as observed via TUNEL staining of apoptotic cells. Statistical significance levels - * p<0.033, ** p<0.0021, *** p<0.0002, **** p<0.0001.

To quantify injury due to different ischemic durations and perfusate composition, as well as correlation with 3RMR, we looked at clinical markers of injury such as portal resistance, ALT & AST hemolysis for pRBC perfusate, alanine aminotransferase (ALT) & aspartate aminotransferase (AST) levels, potassium, and histological markers of injury during and at the end of 3 hours of perfusion for all groups. We observed a higher portal resistance after 3 hours in the packed RBC groups (> 0.01 mmHg*min/ml/g) compared to acellular groups (< 0.01 mmHg*min/ml/g), however, only reached significance for the 0hCI acellular vs 24hCI pRBC groups with p = 0.0086 (Fig. 3a). ALT and AST levels are considered important markers of liver injury, especially during machine perfusion^35^. Their levels were also higher for the 0hCI pRBC and 24hCI pRBC groups, compared to the 0hCI acellular and 24hCI acellular groups at the beginning (ALT: 10, 11 ± 6.557, 0, and 0 U/L; AST: 32 ± 4, 34 ± 9.17, 3 ± 3.83, and 9.33 ± 8.37 U/L) as well as the end (ALT: 27.3 ± 12.7, 51.33 ± 18.037, 4 ± 3.65, and 4 ± 3.46 U/L; AST: 114, 169.3 ± 65.187, 23 ± 12.91, and 40.67 ± 11.015 U/L) of 3 hours of perfusion. The absolute values of ALT and AST for all the groups of livers are shown in Figs. 3b and 3c, respectively. Finally, hyperkalemia (high potassium) is also a marker of injury to the organ that measures release of potassium into the perfusate due to cell death. The potassium concentration stayed between 4–8 mmol/L for all groups during perfusion. Neither drastic shifts over time, nor significant differences between groups at each time were observed as shown in Fig. 3d.

Hemolysis is a marker of injury to RBCs that affects oxygenation capacity as well as microcirculatory resistance of the organs, as the RBC in the perfusate are continuously exposed to mechanical stresses. It may cause endothelial dysfunction and increased resistance to flow in the capillaries^36^. Thus, we quantified hemolysis by analyzing the absorbance properties of the perfusate at 414 nm wavelength (detailed protocol in methods section). We observed increasing hemolysis with the duration of perfusion in both 0hCI pRBC and 24hCI pRBC groups as seen in Fig. 3e, where the absorbance of 414 nm wavelength of light increases at a steady rate over time. The increasing hemolysis can also be observed from the increasing intensity of red coloration of the perfusate, where RBCs have been centrifugally separated (as shown in Fig. 3f). Furthermore, an analysis of histological staining using hematoxylin & eosin (H&E) stain, as well as terminal deoxynucleotide transferase (dUTP) nick end labeling (TUNEL) followed by a blinded analysis by a pathologist was performed. It indicated higher endothelial damage in the 24hCI-pRBC group of livers (as seen in the predominant TUNEL staining of endothelial cells) compared to 24hCI-acellular group where higher damage to hepatocytes (as seen in the predominant TUNEL staining of hepatocytes) was observed. Representative histological micrographs are shown in Fig. 3g. Comparatively, only marginal cell death was observed in the 0hCI-acellular and 0hCI-pRBC groups, indicating sufficient recovery of organs in these groups of livers. Other functional metrics, blood gases, and chemistries were also measured including pH, bile production, weight gain, Ca^++^, Na^+^, and HCO_3_, and are summarized in Supplementary Fig. 1. We observed significantly lower pH in the 24hCI pRBC group of livers (p < 0.0001). We also observed significantly higher Na^+^, but significantly lower Ca^++^ in the pRBC groups. Interestingly, there was no statistical difference in bile production, indicating full recovery of function in all livers. However, the 24hCI livers perfused with both compositions gained weight, indicating the presence of endothelial injury leading to edema, especially in the 24hCI pRBC group of livers.

## Discussion

Solid organ transplantation is a lifesaving procedure, yet severely limited in its capacity to save lives due to ischemia-reperfusion injury (IRI) related complications. Machine perfusion (MP) allows minimization of and recovery from IRI when compared to the current clinical standard of static cold storage. Different machine perfusion methods use different temperatures and perfusate compositions. However, the direct impact of various machine perfusion protocols on mitochondrial injury are incompletely understood. To further enhance the understanding of organ quality during machine perfusion and to increase the overall number of transplants by better assessment of marginal organs, there is a significant push to develop highly specific, sensitive, and quantifiable markers to understand organ health during perfusion. Such technologies help further optimize conditions of perfusion, may provide a better understanding of the underlying mechanisms of IRI, and identify new therapeutic interventions to overcome mitochondrial injury.

We present a novel platform for non-invasive, real-time assessment of oxygenation of mitochondria during *ex vivo* liver perfusion using resonance Raman spectroscopy. Through experiments in rat livers, we aimed to provide mechanistic insights into oxygen demand and supply at the electron transport chain during normothermic machine perfusion-based recovery from cold ischemia. For instance, when a minimally ischemic (< 15 minutes) rat liver is perfused with either an acellular perfusate or packed RBC based perfusate, 3RMR is low indicating sufficient oxygenation (Fig. 2b). However, when a 24-hour cold ischemic (24hCI) liver is perfused, the pRBC perfusate group shows a low 3RMR (< 25, sufficiently oxygenated) while the acellular perfusate group shows a high 3RMR (> 40, insufficiently oxygenated) (Fig. 2c), which is also corroborated by significant hepatocyte death as observed via TUNEL staining. It is likely that there is an accumulation of electrons at the mitochondrial cytochromes during cold ischemia, which can be rapidly transferred to the abundant oxygen molecules in case of pRBC based perfusate. However, this process is comparatively slower in the case of acellular perfusates due to a lower concentration of oxygen. This trend is also supported by the high lactate levels and high oxygen uptake rate in the 24hCI groups immediately upon reperfusion (Figs. 2e and 2f).

While sufficient reoxygenation after ischemia is necessary for recovery, a sudden temperature change and oxygen burst may also exacerbate IRI. Indeed, this suspicion is confirmed by the injury markers ALT and AST, which show higher injury in organs that are perfused with pRBC compared to the organs perfused with an acellular perfusate (Figs. 3b and 3c). Quite interestingly, a marginal increase in 3RMR at the end of the perfusion for the 24hCI pRBC group is also observed without achieving statistical significance (3RMR of 30–40), indicating the possibility of rising injury over time. This damage is also confirmed by histology with TUNEL, where higher endothelial cell death in livers perfused with packed RBCs is observed, as compared to acellular perfusate. It is possible that such a response is triggered by the innate coagulability of pRBCs^39^, RBC damage due to hemolysis (as a result of mechanical stresses in the perfusion circuit) leading to endothelial dysfunction, vasculopathy and reduced bioavailability of nitric oxide^36^, or hyperoxygenation induced breakdown of NO leading to vasoconstriction^40^. Alternatively, it may be hypothesized that the higher supply of oxygen that is available upon reperfusion with pRBCs may lead to ROS generation and reperfusion injury^38^.

Despite these initial high values, the 3RMR for 24hCI-acellular group decreases gradually until about 90 minutes of reperfusion when it becomes comparable to 3RMR in 24hCI-pRBC group as shown in Fig. 2c. It is likely that the accumulated electrons are lowered to physiologic levels by 90 minutes due to the continuous supply of oxygen in the perfusate, albeit at a slower rate compared to the 24hCI pRBC group and are available for ATP production promoting energetic recovery which is an essential criterion to accept organs for transplant. This is confirmed by the energy charge values and NAD:NADH ratios (Fig. 2g) which were statistically similar in all groups at the end of 3 hours of NMP. Furthermore, lactate levels were also similar for most of the duration of perfusion, indicating that all livers likely remain transplantable including those in the acellular perfusate group.

3RMR may indeed provide an opportunity for dynamic optimization of recovery of livers, by real-time optimization of temperature and perfusate conditions during machine perfusion. For instance, further optimization of the recovery of cold stored organs using MP before transplant may involve a controlled rate of increasing the temperature of the organ after cold storage. Such controlled increase in temperature may slow down the recovery of electron transport chain function, and thus avoid the higher stress associated with sudden change in temperature and ROS generation upon reperfusion. Indeed, in our previous study, when 24hCI livers were recovered with acellular perfusate using sub-normothermic machine perfusion (SNMP), we observed that the 3RMR levels falls to low level within 30 minutes of perfusion^37^. This may be explained by a slower rate of accumulation of electrons at the complexes III and IV at sub-normothermic temperatures compared to the normothermic temperatures due to the relatively slower rate of electron transport chain activity at this temperature. A slower push and pull of electrons at this temperature in the initial phase of perfusion is likely to be favorable for the overall health of the mitochondria and lead to overall reduced stress on the electron transport chain while allowing ATP production and better functioning of repair mechanisms.

In conclusion, there is an urgent need for mechanistic, non-invasive, and real-time assessment of IRI during machine perfusion due to the low specificity and sensitivity of currently used markers^41^. We successfully developed a novel approach that provides a reporter free highly specific readout of functional oxidation state of mitochondrial cytochromes during machine perfusion in real-time. Better understanding of mitochondrial injury may not only help in better optimization of storage and perfusion conditions, but also assess the extent of injury and function of organs before transplant^42^. Thus, optimal assessment may allow the use of organs from donors deceased due to cardiac death (DCD) and other extended criteria donor (ECD) organs. This will help increase the number of organs available for transplant and improve the quality of organs that are transplanted leading to an overall improved standard of care for patients requiring this life saving treatment. Based on our observations of 3RMR at different temperatures (SNMP and NMP), perfusates (acellular and pRBC perfusate), and durations of storage (fresh, 24-hour cold storage, 72-hour cold storage) in rat livers, we observe that ischemic livers have a higher demand for oxygen at the beginning of perfusion, yet packed RBCs may exacerbate liver damage by the end of machine perfusion.

## MATERIALS AND METHODS

### Liver procurement and storage

All animals for the experiments were maintained in accordance with National Research Council guidelines and were approved by the Institutional Animal Care and Use Committee (IACUC) at Massachusetts General Hospital (Boston, MA, USA). Female Lewis rats (200g, Charles River Laboratories) were sedated in an induction chamber using isoflurane set to 3–5%. The excess vapor was filtered through with activated charcoal (Vet Equip Vapor Guard Activated Charcoal, 931401). The animal was then removed from the chamber and placed on a surgical table in a supine position. Depth of anesthesia was deemed adequate when muscular contraction was absent following toe pinch. The abdominal region was shaved, and abdomen is opened with a transverse abdominal incision. The hepatic artery and branches of portal vein were ligated and 200U of sodium heparin (MGH Pharmacy) was injected into the supra-hepatic vena cava. The bile duct was cannulated with a 22G polyethylene tube and a 16G catheter (BD Insyte Authoguard, 381454) was inserted into the portal vein, and the IVC transected. The liver was immediately flushed with 40mL of heparinized saline solution and an additional 20mL to remove any residual blood. Perfusion was started within 5 minutes of harvesting, except for livers exposed to durations of static cold storage events which were further flushed with 25mL of chilled University of Wisconsin and stored in 50mL of UW solution at 4°C.

### Normothermic machine perfusion setup

The perfusion set-up provides oxygenated continuous flow through the portal vein at temperature of 37°C. The specifics of the machine perfusion protocol used were previously covered elsewhere^43^. Perfusion was pressure and flow controlled; the flow gradually increased from 6 ml/min to a maximum perfusion rate of 30 ml/min.

Acellular perfusate was composed of base William’s E medium containing 1.022g of BSA (Sigma-Aldrich, A7906), 2.4mg of dexamethasone (Sigma-Aldrich, D2915), 1.02mL of penicillin streptomycin (Sigma-Aldrich, P4458), 1.02mL of Glutamax (Thermo Fisher Scientific, 35050061), 0.5U of insulin and 200U of heparin. Perfusate was sterile filtered, and pH stabilized at 7.4 by adding sodium bicarbonate (MGH Pharmacy). For the cellular perfusate group, 80mL of acellular perfusate was added to 20mL packed red blood cells (hematocrit 15–20%) to bring total volume up to 100mL. Prior to perfusion of the liver, the perfusate was cycled for about 15 minutes in the perfusion system to oxygenate and warm it to 37°C.

### Red blood cell collection and preparation of cellular perfusate

The donor rats were anesthetized by inhalation of isoflurane (3–5%) in an induction chamber and depth of anesthesia was confirmed by lack of response following toe pinch. The animal was then placed on heating pad in a supine position, and the area around the lower rib was shaved. A small midline incision was made, and the underlying tissue was separated. The heart was located and a 23G needle attached to a 10mL syringe containing xml of sodium heparin was inserted into the left ventricle, and the slowly retracted until no blood was available. A total of 6–8mL of blood was collected and the rat was euthanized by exsanguination.

Whole blood collected in sodium heparin was spun down at 2.200 gm for 10 mins at 20°C. The plasma and buffy coat layers are aspirated, and red blood cells are resuspended in perfusate and spun down two more times for the wash process. The washed RBCs are transferred into sterile storage tubes and stored at 4°C for up to 5 days.

### Resonance Raman Spectroscopy Setup

The portable RRS System (Pendar Technologies) was developed as a compact 441 nm laser device housing a power laser source of 8.9mw and compact probe with a flexible fiber optic bundle. The probe head is an 8mm × 12mm × 60mm which delivers single mode laser light about 1.5 mm diameter on the tissue. The light is passed through the collection optics, and the filtered RR photons are collected at the temperature-controlled two-dimensional charge coupled device (CCD) detector array. Measurements are recorded for total time of 180s with isolated signals from mitochondrial in the 700–1700 cm^−1^ spectral range. The readout from the CCD is recorded and analyzed using software developed in LabView.

### Experimental Design

Our experimental groups that consist of livers with low and high demand of oxygen (0hCI and 24hCI livers) were perfused with perfusates carrying a low and high supply (acellular and pRBC perfusate) of oxygen as summarized in Fig. 2a. For each of these groups, we obtained the 3RMR value every 30 minutes starting roughly 5 minutes after the beginning of machine perfusion. We also performed an analysis of the blood gases and chemistry of the perfusate at every hour of perfusion to validate the perfusion technique. At the end of the perfusion, wedge biopsies were flash frozen for energetic analysis including ATP, ADP, AMP ratios, and NAD:NADH ratio that show mitochondrial electron transport chain activity both downstream (i.e., at complex V) and upstream (i.e., at complex I) of 3RMR. Finally, we also compared injury to the graft using markers including flow resistance during perfusion, hemolysis, potassium level, ALT/AST levels every 60 minutes during perfusion, and histological markers of injury at the end of perfusion.

### Perfusion data acquisition and processing

Blood gas analysis by perfusate sampling every thirty minutes using the RAPIDPOINT 500 Blood gas analyzer (Siemens Healthineers, Munich, Germany) for measurements of blood gases and chemistries including pH, pO2, electrolytes (Na^+^, K^+^, Ca^++^, CI^−^), and metabolites (lactate). AST and ALT concentrations were measured hourly from the venous outflow using the Piccolo Xpress Chemistry Analyzer (Abbott, Illinois, USA). For hematological analysis, perfusate samples collected were centrifuged at 4000g for 10 mins. Supernatant were collected and stored at − 20°C which were later recovered for free hemoglobin assessment using the NanoDrop One Microvolume UV-Vis Spectrophotometer (ThermoFisher Scientific, Waltham, MA, USA) at 414nm. Hematocrit levels were checked prior to perfusion using the Sysmex XP-300^™^ Automated Hematology Analyzer (Sysmex, Kobe, Japan).

The liver was weighed immediately after harvesting and after machine perfusion. The pressures and flow rates were recorded every 30 mins during the 3 hours perfusion run. Liver tissues were flash frozen in liquid nitrogen immediately after perfusion and stored at −80°C. The concentrations of NAD, NADH, NADP and NADPH in liver tissue were analyzed by mass spectrometry core of Shriners’ Children’s Boston (Boston, MA, USA). Liver biopsies were also fixed in 10% formaldehyde for a maximum of 96hrs before transferring into 70% ethanol. Fixed tissues were processed for TUNEL and H&E staining, which was performed by the Massachusetts General Hospital Histology Molecular Pathology Core (Charlestown, MA, USA).

### Statistical Analysis

All statistical analyses were performed in Prism 9 (GraphPad Software Inc., La Jolla, CA). Two-way ANOVA based multiple comparison tests in GraphPad were used to derive statistical conclusions.

## Data Availability

The authors affirm that the primary data supporting the conclusions of this research are represented within the article. Any other information not contained within the article will be made available by the corresponding author upon reasonable request.
